# A Single Mutation in the VP1 Gene of Enterovirus 71 Enhances Viral Binding to Heparan Sulfate and Impairs Viral Pathogenicity in Mice

**DOI:** 10.3390/v12080883

**Published:** 2020-08-13

**Authors:** Xianliang Ke, Yuan Zhang, Yan Liu, Yuanjiu Miao, Caishang Zheng, Dan Luo, Jianhong Sun, Qinxue Hu, Yi Xu, Hanzhong Wang, Zhenhua Zheng

**Affiliations:** 1 The Joint Center of Translational Precision Medicine, Guangzhou Institute of Pediatrics, Guangzhou Women and Children’ss Medical Center, Guangzhou 510623, China; xianke909@163.com; 2 The Joint Center of Translational Precision Medicine, Wuhan Institute of Virology, Chinese Academy of Sciences, Wuhan 430071, China; 3 CAS Key Laboratory of Special Pathogens and Biosafety, Center for Emerging Infectious Diseases, Wuhan Institute of Virology, Chinese Academy of Sciences, Wuhan 430071, China; zhangyuan@wh.iov.cn (Y.Z.); liuy@wh.iov.cn (Y.L.); miaoyuanjiu@163.com (Y.M.); z-cshang@163.com (C.Z.); luodan64@126.com (D.L.); sunjianhong0604@sina.com (J.S.); qhu@wh.iov.cn (Q.H.); wanghz@wh.iov.cn (H.W.); 4 Guangzhou Women and Children’s Medical Center, Guangzhou Medical University, Guangzhou 511483, China

**Keywords:** enterovirus 71, adaptive mutation, virulence, heparan sulfate, attenuation, VP1-98

## Abstract

Enterovirus 71 (EV71) is the major causative pathogen of human hand, foot, and mouth disease (hHFMD) and has evolved to use various cellular receptors for infection. However, the relationship between receptor preference and EV71 virulence has not been fully revealed. By using reverse genetics, we identified that a single E98K mutation in VP1 is responsible for rapid viral replication in vitro. The E98K mutation enhanced binding of EV71-GZCII to cells in a heparan sulfate (HS)-dependent manner, and it attenuated the virulence of EV71-GZCII in BALB/c mice, indicating that the HS-binding property is negatively associated with viral virulence. HS is widely expressed in vascular endothelial cells in different mouse tissues, and weak colocalization of HS with scavenger receptor B2 (SCARB2) was detected. The cGZCII-98K virus bound more efficiently to mouse tissue homogenates, and the cGZCII-98K virus titers in mouse tissues and blood were much lower than the cGZCII virus titers. Together, these findings suggest that the enhanced adsorption of the cGZCII-98K virus, which likely occurs through HS, is unable to support the efficient replication of EV71 in vivo. Our study confirmed the role of HS-binding sites in EV71 infection and highlighted the importance of the HS receptor in EV71 pathogenesis.

## 1. Introduction

Enterovirus 71 (EV71) is a member of the *Enterovirus* genus in the *Picornaviridae* family, and it has caused repeated outbreaks of human hand, foot, and mouth disease (hHFMD) in children in recent decades [1]. Compared to the other enterovirus members, EV71 is responsible for the most severe HFMD cases and is recognized as one of the most important neurological pathogens [1].

EV71 infects host cells directly by binding to various receptors on the cell surface [2,3]. Among the known receptors, SCARB2 not only mediates cellular attachment and internalization, but also initiates the uncoating of EV71 in low pH conditions [4,5], indicating that it is an uncoating receptor. Other proteins including P-selectin glycoprotein ligand-1 (PSGL-1) [4,6], annexin II [7], nucleolin [8], vimentin [9], and fibronectin [10] are classified as attachment receptors because no evidence of uncoating functions has been found. In addition, heparan sulfate (HS), a highly sulfated glycosaminoglycan composed of repeating disaccharide units, also binds to EV71 and improves virus infection [11]. The above-described receptors bind directly to the capsid of EV71, and several residues in the EV71 structural proteins have been documented to determine receptor preference, viral growth kinetics in vitro and virulence in vivo [12,13,14,15].

We previously identified the highly virulent mouse EV71 strain EV71-GZCII from clinical samples [12]. In this study, EV71-GZCII was adapted to the L929 mouse fibroblast cell line by serial passages, and the EV71 GZCII-P30 novel strain was generated, which showed enhanced replication in infected cells. The E to K mutation at the VP1-98 residue was mainly responsible for the improved growth kinetics of EV71 GZCII-P30 in vitro in an HS-dependent manner. However, GZCII-98K-infected mice showed minimal signs of disease and histopathological features. These findings support previous findings of HS-binding sites on EV71 [14,16,17] and indicate that the HS-binding capability may significantly affect the pathogenicity of EV71 in vivo.

## 2. Materials and Methods

### 2.1. Cells and Viruses

RD (human rhabdomyosarcoma) and Vero (monkey kidney) cells were cultured in Minimum Essential Media (MEM) supplemented with 10% Fetal Bovine Serum (FBS). L929 (mouse fibroblast), Neuro2a (mouse neuroblastoma), and HeLa (human cervical epithelioid carcinoma) cell lines were maintained in Dulbecco’s Modified Eagle Medium (DMEM) supplemented with 10% FBS. EXT1- or SCARB2-knockout cell lines and recombinant viruses were generated as described in the Supplementary Materials. Cells were incubated with virus (diluted in DMEM) for one hour. After washing twice, DMEM with 2% FBS was added (which was set as time point zero). The virus titer at different time points were determined in Vero cells using the Reed and Muench method [18].

### 2.2. Virus Passage for Adaptation

L929 cells were infected with EV71-GZCII (Multiplicity of infection or MOI = 1). At 72 h post-infection, 500 μL of the supernatant was used to infect pre-seeded L929 monolayers without titering. The passage procedure was repeated thirty times. Viruses after each passage were preserved for analysis.

### 2.3. Reverse Genetics and Recombinant Virus Rescue

An infectious clone of EV71-GZCII was constructed by replacing the virus genomic region on pEV71-BrCr [19] with that of EV71-GZCII through homologous recombination-based cloning. Briefly, cDNA was synthesized from reverse transcription of virus genomic RNA using SuperScript III Reverse Transcriptase (Invitrogen) with random primers. Then, two individual polymerase chain reactions (PCR) were performed to amplify the 1–3400 and 3400–7526 nucleotides of viral cDNA, and a third was performed for the vector backbone of pEV71-BrCr. The primers used were designed to produce fragments containing a 15 bp overlapping region at both ends for homologous recombination with each other. Purified DNA fragments were treated with the ClonExpress^®^II One Step Cloning Kit (Vazyme Biotech, Jiangsu, China) and transformed into competent *E. coli* cells to generate the infectious clone pEV71-GZCII. For site-directed mutagenesis, DNA fragments carrying 15-bp overlap sequences for recombination were amplified using KOD-Plus-Neo (Toyobo, Osaka, Japan) with primers carrying desired mutations and pEV71-GZCII as the template. Then, a homologous recombination-based cloning procedure was performed as described above. For recombinant virus rescue, infectious clone plasmids with mutations were cotransfected into Vero cells with the T7 polymerase-encoding plasmid pT7 using Lipofectamine 3000 (Life Technologies, Carlsbad, CA, USA). Cells were subjected to three freeze-thaw cycles after an obvious cytopathic effects (CPE) appeared. Supernatants were filtered through a 0.45 μm membrane and stored at −80 °C. Primers are listed in Table S1.

### 2.4. CRISPR Knockout of the SCARB2 and EXT1 Genes

sgRNAs were designed using the E-CRISP online tools [20]. For sgRNA-expressing plasmid construction, DNA oligos were constructed into LentiCRISPRv2 (Addgene #98290), according to the manual. For lentivirus production, the packaging plasmids PsPAX2 (#12260) and pMD2G (#12259) were used and the constructed lentiCRISPRv2-sgRNA plasmid was transfected into HEK293T cells at a ratio of 6:3:1 using a calcium phosphate transfection reagent (Promega, Madison, WI, USA). Supernatants collected at 48 h post-transfection were filtered through a 0.45 µm filter and used directly without storage. Cells infected with CRISPR-Cas9 lentiviruses were selected with puromycin (10 μg/mL for L929 cells) for one week. Then, single cell clones were picked through serial dilution of the puromycin-resistant cells in 96-well plates. For genotyping, the genome region of the cell clones containing the sgRNA target sequence was amplified, subcloned, and subjected to Sanger sequencing. SCARB2 expression was detected through western blotting using a rabbit polyclonal antibody against SCARB2 (ProteinTech, Rosemont, IL, USA). HS expression on wild-type and EXT1-knockout L929 cells was detected by flow cytometry using a mouse IgM antibody against HS (F58-10E4, AMSBIO) as the primary antibody.

### 2.5. Next-Generation Sequencing (NGS)

cDNA of non-passaged GZCII virus and GZCII-P30 was synthesized using the Superscript III First Strand Synthesis Systems (Invitrogen, Carlsbad, CA, USA). Sequence-specific PCR was performed using KOD-Plus-Neo polymerase (Toyobo, Osaka, Japan) to produce two DNA fragments covering the entire viral genome. Amplicons were pooled to generate libraries and were subjected to the HiSeq 3000 system for sequencing. After cleaning, the reads of EV71-GZCII or GZCII-P30 were mapped to the pEV71-GZCII sequence. Single nucleotide polymorphisms (SNPs) were called in CLC Genomics Workbench Version 7 using a frequency threshold of 1%.

### 2.6. Cell-Binding Assay

Cells were detached using 0.5% Edetate disodium (in Phosphate-Buffered Saline or PBS, pH 7.2) and counted using an automated cell counter (Thermo Fisher, Waltham, MA, USA). Cell suspensions were then aliquoted, washed twice with PBS and collected by centrifugation at 2000 rpm and 4 °C for 5 min. Viruses were diluted to a final concentration of 10^6^ TCID_50_/mL. Diluted viruses (200 μL) were preserved as input samples, and the remaining viruses were used to resuspend cells. The mixtures of cells and viruses were kept at 4 °C for 1 h and then centrifuged at 2000 rpm and 4 °C for 5 min. Total RNA of the L929 cells was subjected to EV71-specific real time Quantitative Reverse Transcription PCR (qRT-PCR) to determine the viral copy number and mouse Glyceraldehyde-3-Phosphate Dehydrogenase (GAPDH)-specific qPCR was used to normalize the cell number. The virus-binding activity was evaluated by determining the ratio of attached virus to the total input virus, followed by normalization to the GAPDH expression level.

### 2.7. Heparin Sepharose Binding Assay

Different viruses (10^6^ RNA copies) were loaded onto a 1 mL prepacked gravity Heparin sepharose column (Sangon Biotech, Shanghai, China) for binding. Upon virus loading, columns were washed six times with 5 mL of washing buffer (0.02 M Tris, pH 7.5; 0.14 M NaCl). Attached viruses were eluted using 5 mL of elution buffer (0.02 M Tris, pH 7.5; 2 M NaCl). Input and eluted viruses were subjected to qRT-PCR to quantify the binding efficiency.

### 2.8. Heparin Inhibition Assay

GZCII, GZCII-P30, cGZCII, and cGZCII-P30 viruses were mixed with 500 μg/mL soluble heparin salt at 37 °C for 1 h as previously described [11]. Pretreated viruses were incubated with L929 cells seeded in 24-well plates. Cells were washed twice and cultured for 24 h. Virus production and viral RNA levels were detected using the TCID_50_ method and qRT-PCR, respectively.

### 2.9. Mouse Infection and Viral RNA Copy Number Determination

All mouse experiments were reviewed and approved by the Animal Care Committee of the Wuhan Institute of Virology (permit no. WIVA07201606). Two-week-old BALB/c mice were intraperitoneally inoculated with EV71-GZCII variants at different dosages. Mouse survival and disease development were observed for at least 15 days. Disease development was scored as previously reported [12]. To determine viral replication in vivo, blood was collected from the facial vein of the infected mouse. Mouse tissues including liver, lung, kidney, heart, skeletal muscle, intestine, spleen, and brain, were harvested under anesthesia. Blood and mouse tissues were homogenized in TRIzol Reagent (Invitrogen, USA) using zirconia beads. Viral RNA copy number in 1 μg of total RNA was used to evaluate the viral replication level.

### 2.10. Mouse Tissue Homogenate Binding Assay

Freshly harvested mouse liver, brain, kidney and intestine tissues were homogenized in ice-cold PBS (0.1 M, pH 7.2). Insoluble fractions were separated by centrifugation at 3000 rpm for 10 min at 4 °C, washed twice, and then used for the virus-binding assay. GZCII, GZCII-P30, cGZCII, and cGZCII-98K viruses were diluted to ~10^6^ copies/mL in DMEM (measured using qRT-PCR). Dilutions (150 μL) were stored for quantification of the input virus amount, and the remaining viruses were incubated with the tissue homogenate for attachment. After attachment, the mixture was centrifuged at 3000 rpm for 10 min at 4 °C, and the supernatant was collected. Virus attachment efficiency was evaluated by determining the adsorbed/input virus ratio.

### 2.11. Immunohistochemistry

Paraffin-embedded sections of brain, liver, lung and intestine tissues from BALB/c mice were immunostained to evaluate the expression and distribution of HS and SCARB2. Sections were heated, deparaffinized with xylene and then cleared with alcohol. Following antigen retrieval, slides were blocked with 3% normal goat serum in PBS for 1 h. Subsequently, a rabbit anti-SCARB2 antibody (ProteinTech; 1:200 dilution) and a mouse anti-HS IgM (Amsbio, Abingdon, UK; 1:200 dilution) antibody, which can detect various kinds of HS, were incubated with the sections overnight at 4 °C. FITC-conjugated goat anti-rabbit IgG (ServiceBio, Hubei, China) and Cy3-conjugated goat anti-mouse IgM (ServiceBio, Hubei, China) antibodies were subsequently incubated with sections for 1 h at 37 °C. Images were acquired using a Panoramic scanner (3D-Histech, Hungary).

### 2.12. Statistics

Student’s *t*-test was performed for all in vitro studies. At least two biological replicates were included for each experiment. GraphPad Prism 6.0 (GraphPad Software, San Diego, CA, USA) was used for statistical analyses, and a *p* value < 0.05 was considered significant.

## 3. Results

### 3.1. Production and Characterization of an L929-Adapted Virus

**Figure 1:**
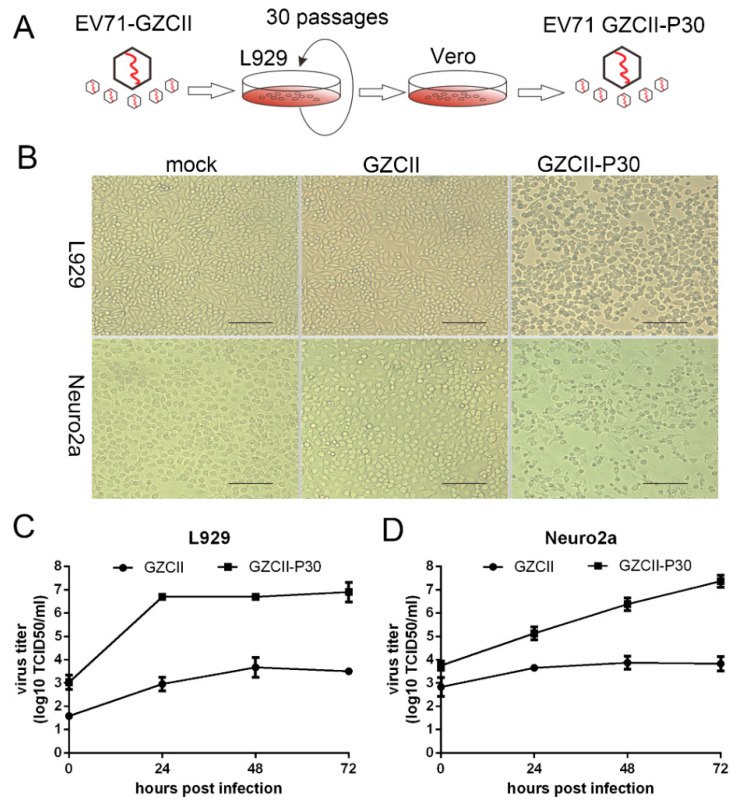
Generation and in vitro characterization of the L929-adapted EV71-GZCII virus. (**A**) EV71-GZCII virus was passaged 30 times in L929 cells to generate the GZCII-P30 strain. (**B**) L929 and Neuro2a cells were infected with GZCII or GZCII-P30 (MOI = 0.1) or mock infected for 24 h. Scale bar: 200 μm. (**C**) Multistep growth curve of GZCII and GZCII-P30 in L929 and Neuro2a (MOI = 1) cells (**D**). Viral titers were determined by measuring TCID_50_.

EV71-GZCII effectively infected 2-week-old mice and replicated to produce high titers in multiple mouse tissues [12], suggesting that EV71-GZCII likely has the capability to replicate in murine cells. To address this possibility, L929 and Neuro2a cells were used for an EV71-GZCII virus infection. The viral titer in both murine cell lines increased as the infection time was prolonged (Figure 1C,D). However, no obvious cytopathic effect (CPE) was observed (Figure 1B). This indicates either a lower replication rate or a non-lytic exit mechanism [21,22] was employed by this virus.

To test whether adaptive passages of L929 cells could improve the fitness of EV71-GZCII in murine cells, we passaged the EV71-GZCII virus in L929 cells for 30 generations and generated an adapted strain named EV71 GZCII-P30 (Figure 1A). EV71 GZCII-P30 showed distinguished cytotoxicity and growth kinetics in both murine cell lines (Figure 1). Massive cell death was observed in L929 and Neuro2a cells infected with the GZCII-P30 virus, but not with the GZCII parental strain (Figure 1B). Growth curves showed a substantial increase in the virus titer with GZCII-P30 compared to with GZCII (Figure 1C,D), which may be the reason for their differences in causing a CPE in L929 and Neuro2a cells. These results demonstrate that GZCII-P30 is highly adaptive during serial passage in vitro.

### 3.2. Sequence Analysis Reveals Multiple Mutations between Adapted and Parental Viruses

**Figure 2:**
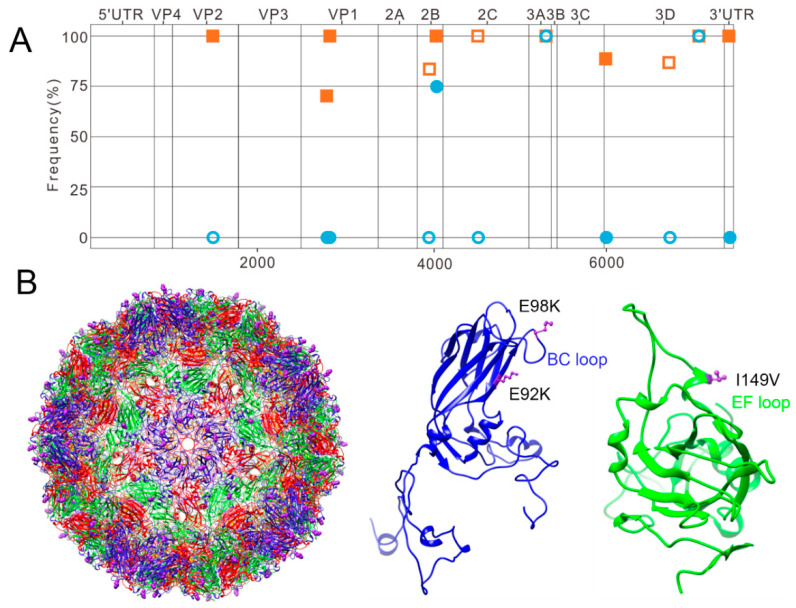
Adaptive mutations in the GZCII-P30 genome revealed by Next-Generation Sequencing were mapped onto the EV71 capsid. (**A**) Genomic RNA of the GZCII and GZCII-P30 viruses was sequenced by NGS, and the mutation frequency and distribution were analyzed according to the reference sequence (cGZCII). The open symbols represent nonsynonymous mutations, and the closed symbols are synonymous mutations. Red: GZCII-P30; Blue: GZCII. (**B**) A 3D structure of the GZCII-P30 virus was predicted by SWISS-MODEL homology modeling based on PDB:3VBF.1. VP1-E92K and E98K reside at the 5-fold axis, while VP2-I149V is located in the E-F loop on VP2. Blue: VP1; red, VP2; green, VP3; yellow, VP4.

NGS analysis was performed to detect mutations between the GZCII-P30 virus and the GZCII origin strain. In total, 12 mutations were revealed by alignment including six synonymous and five nonsynonymous mutations in the coding region as well as one additional mutation in the 3′UTR of the viral genome (Figure 2A).

Protein sequence alignment revealed three mutations in structural proteins (VP2-I149V, VP1-E92K, and VP1-E98K) and two mutations in nonstructural proteins (2B-N45S and 3D-K7R) (Figure S1A). The frequencies of the three mutations in the viral capsids were 99.7% for VP2-I149V, 70.1% for VP1-E92K, and 99.9% for VP1-E98K (Figure 2A). Mapping the mutations on the 3D viral structure showed that two mutations on VP1 were located near the 5-fold axis of the EV71 capsid (Figure 2B). E98K was exposed at the top of the loop sequence, while E92K was less exposed, but still adjacent to the viral surface. The VP2-I149V mutation was located near the canyon region of the viral capsid but was also exposed on the E-F loop of VP2 (Figure 2B).

### 3.3. E98K on the Viral Capsid Is Mainly Responsible for Accelerated Replication in Infected Cells

**Figure 3:**
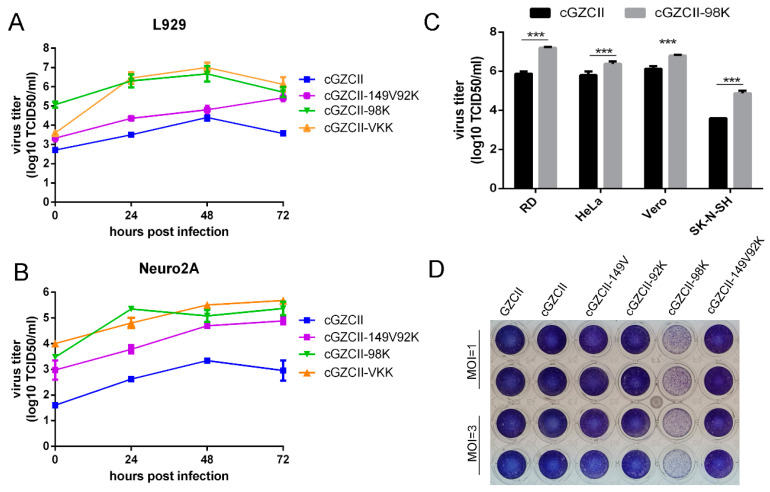
Replication kinetics of the recombinant GZCII viruses in cells. (**A**) Growth curves of GZCII variants in L929 or (**B**) Neuro2a cells (Multiplicity of infection = 0.1). (**C**) Vero, RD, HeLa, and SK-N-SH cells infected with cGZCII or cGZCII-98K virus (MOI = 0.1) were harvested at 24 h post-infection for virus titration in Vero cells (***: *p* < 0.001). (**D**) L929 cells in 96-well plates were infected with GZCII mutants (MOI = 1 or 3), fixed, and stained using crystal violet to assess cell cytotoxicity.

As all three mutations on the viral capsid showed potential effects on receptor binding, we constructed recombinant viruses (Figure S1) to characterize their functions. Compared to the clone-derived GZCII (cGZCII in brief), the cGZCII-98K virus replicated to high titers close to those of the triple-mutated virus strain (cGZCII-VKK). In contrast, the cGZCII-149V92K virus showed moderately increased titers in cells (Figure 3A,B). CPE imaging showed similar results. cGZCII-E98K infected L929 cells showed an obvious CPE in L929 cells. However, cGZCII or recombinant viruses without the VP1-E98K mutation failed to induce a visible CPE (Figure 3D). These results indicate that the E98K mutation on VP1 is mainly responsible for cytotoxicity and improved replication kinetics. Furthermore, the virus titration assay showed that the E98K mutation also facilitated viral replication in RD, Vero, and HeLa cells (Figure 3C), implying that the replication enhancement function of E98K is not species-specific.

### 3.4. VP1-E98K Mutation Alters the Dependency of EV71-GZCII on mSCARB2 for Infection

**Figure 4:**
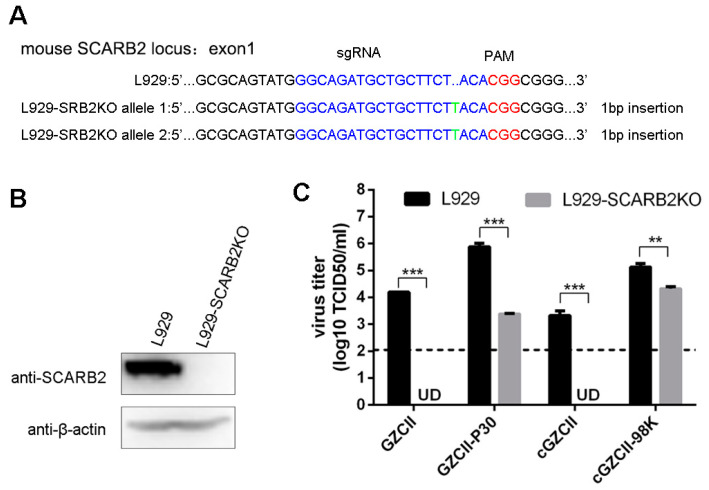
Viral replication in SCARB2-knockout L929 cells. (**A**) Construction of a SCARB2-knockout cell line. Both alleles of L929-SCARB2KO cells were genotyped by Sanger sequencing to confirm SCARB2 gene disruption. (**B**) Expression of SCARB2 in wild-type L929 or L929-SCARB2KO cells was detected by western blotting. GZCII mutants were used to infect L929 and L929-SCARB2KO cells (MOI = 0.1). Viral titer (**C**) at 24 h post-infection were detected by TCID_50_ method. (**: *p* < 0.01; ***: *p* < 0.001).

As mouse SCARB2 (mSCARB2) exhibits only ~85.8% amino acid identity to the human SCARB2 (hSCARB2) protein, most EV71 isolates cannot use mouse SCARB2 to infect L929 cells [23]. We examined the dependency of GZCII variants with different mutations by infecting mSCARB2-knockout L929 cells (L929-SCARB2KO) (Figure 4). The viral titer decreased significantly when mouse SCARB2 was knocked out in L929 cells (Figure 4C). Both GZCII and cGZCII viruses in L929-SCARB2KO cells were below the detection threshold, suggesting a mSCARB2-dependent way of infection. Interestingly, more than 10^3^ 50% tissue culture infectious dose (TCID_50_) units of the GZCII and cGZCII viruses were detected (Figure 4C). The results indicate that while EV71-GZCII infects L929 cells in an mSCARB2-dependent manner, VP1-E98K mutation likely enables virus binding to other receptors.

### 3.5. VP1-E98K Mutation Enhances Virus Binding to Cells via HS

**Figure 5:**
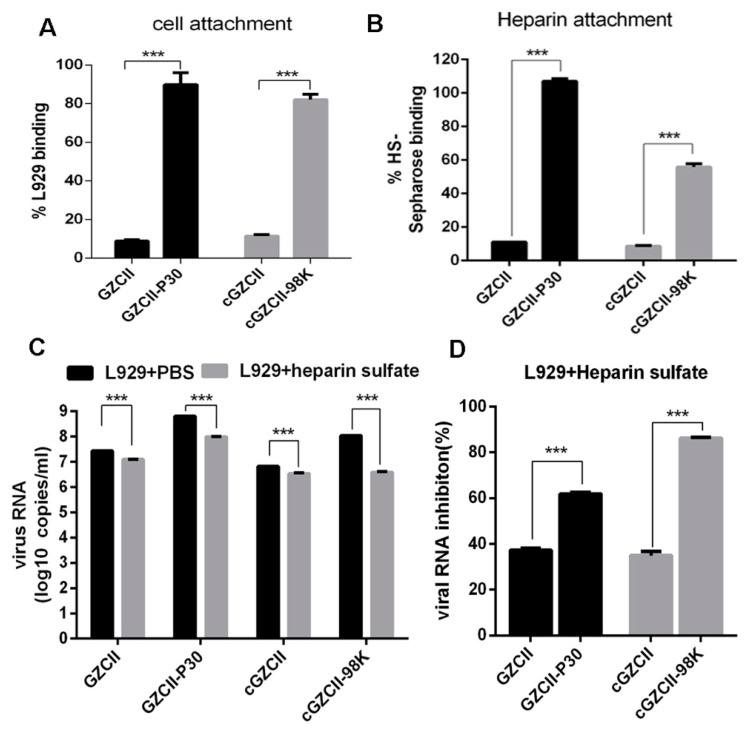
Characterization of the binding efficiency and sensitivity of EV71-GZCII variants to heparin sulfate (HS). (**A**) Viruses were incubated with detached L929 cells (**A**) or an HS-sepharose column (**B**) to allow attachment, and the ratio of adsorbed to input virus was determined by qPCR. Viruses pretreated with soluble HS sodium were used to infect L929 (MOI = 0.1), and the viral RNA copy numbers were determined at 24 h post-infection (**C**). The percentage of viral RNA change is shown in (**D**). (***: *p* < 0.001).

Considering the relatively high titer of 98K-containing viruses at the zero time point in the growth curve assay (Figure 2A,B), we propose that VP1-E98K may enhance the attachment of EV71 to cells in the absence of mouse SCARB2. To address this possibility, 10^6^ RNA copies of GZCII, GZCII-P30, cGZCII, and cGZCII-98K viruses were incubated with 10^6^ L929 cells, and the relative attachment quantity was quantified. As expected, strains carrying E98K mutations (GZCII-P30 and cGZCII-98K) showed an increased percentage of cell binding (Figure 5A).

As the E98K mutation on VP1 has been previously reported for its role in HS binding [14,15], we applied equal amounts of the above four viruses to a heparin sepharose column to compare the binding activities. Both GZCII-P30 and cGZCII-98K showed stronger attachment than the wild-type virus, but the percentage of the captured GZCII-P30 virus was higher than that of the captured cGZCII-98K virus (Figure 5B), indicating that other mutations (VP2-I149V or VP1-E92K) may also contribute to HS attachment. We further tested the role of the HS-binding capability for viral replication. All four viruses pretreated with heparin sodium replicated less effectively than the control group in L929 cells, as depicted by viral RNA copy number detection (Figure 5C). GZCII and cGZCII were also sensitive to HS treatment, but less sensitive than the GZCII-P30 or cGZCII-98K virus (Figure 5D). Together with the result of the heparin and cell binding assay, the inhibition of heparin on virus RNA level may be a result of adsorption prevention.

### 3.6. Depletion of HS through EXT1-Knockout Inhibits Viral Replication In Vitro

**Figure 6:**
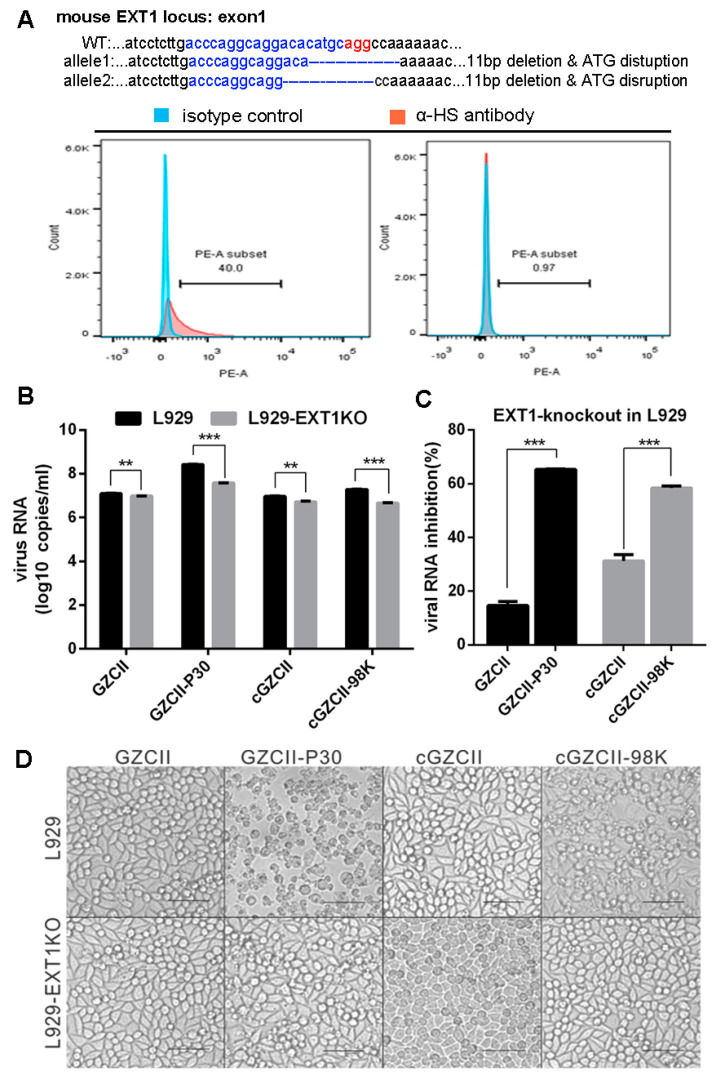
Replication of EV71-GZCII variants in wildtype and EXT1-knockout L929 cells. Cloned L929-EXT1KO cells were genotyped to confirm gene disruption or analyzed by flow cytometry to detect HS expression (**A**). Normal mouse IgM was used as an isotype control. Replication and cytotoxicity of GZCII mutants in HS-depleted cells. L929 and L929-EXT1KO cells (**B**) were infected with GZCII variants (MOI = 0.1). Viral RNA copy numbers and percent inhibition were determined at 24 h post-infection by qRT-PCR. Viral RNA copy numbers and percent inhibition by EXT1KO were determined (**C**). Images of infected wild-type or EXT1 knockout cells were acquired at 24 h post-infection (**D**) to observe the CPE induced by GZCII variants. Scale bar: 200 μm. (**: *p* < 0.01; ***: *p* < 0.001).

HS biosynthesis is catalyzed by multiple enzymes [24]. EXT1, a glycosyltransferase involved in the chain elongation step of HS biosynthesis, can be knocked out to deplete cellular HS [11,25]. We constructed EXT1-knockout L929 cell lines using CRISPR-Cas9 technology to test the dependency of cGZCII variants on HS. Sanger sequencing showed that the start codon of EXT1 alleles was depleted from the cloned cell lines (Figure 6A). HS expression on the cell surface was confirmed by flow cytometry (Figure 6A). As expected, RNA replication of all four GZCII variants was significantly reduced in the EXT1KO cell lines (Figure 6B). GZCII-P30 and cGZCII-98K induced severe cell death of L929 cells, but the CPE was impaired in L929-EXT1KO cell lines (Figure 6D). The percent inhibition of the cGZCII-98K RNA levels was significantly higher than that of cGZCII (Figure 6C).

### 3.7. Cell-Adapted GZCII-P30 Virus Is Attenuated in Mice

**Figure 7:**
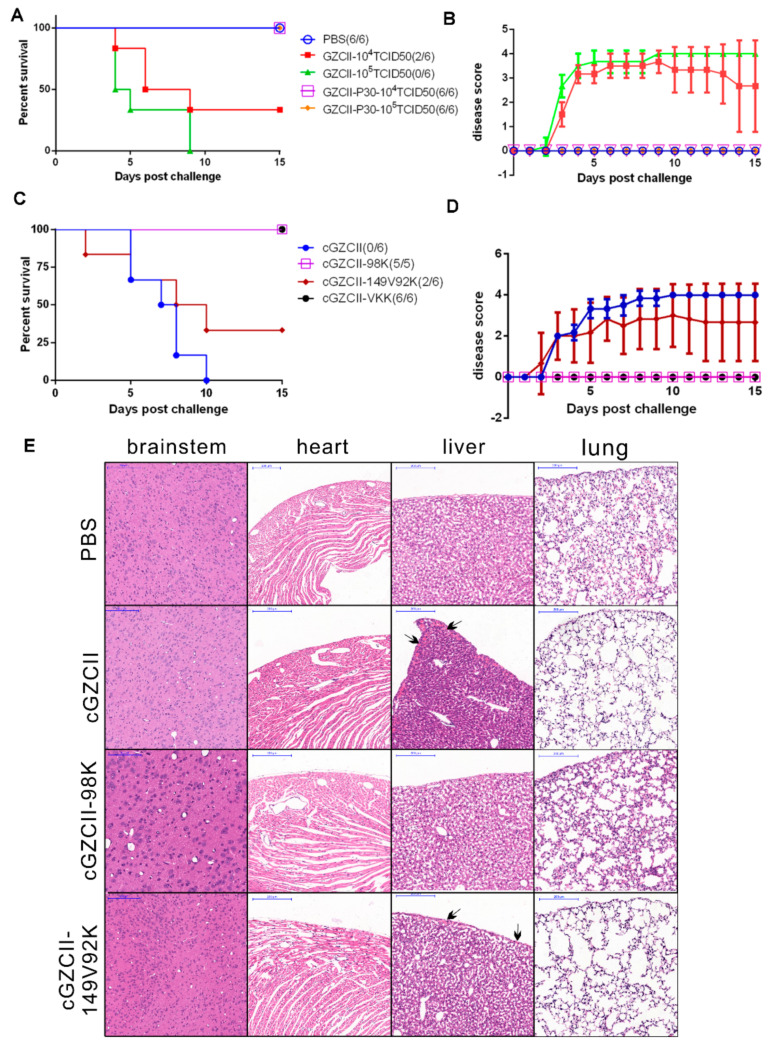
Virulence and pathogenesis of GZCII variants in 2-week-old BALB/c mice. Mice were infected with (**A**,**B**) GZCII or GZCII-P30 virus (10^4^ or 10^5^ TCID_50_ units, respectively, *n* = 6) or (**C**,**D**) cGZCII-98K, cGZCII-149V92K, and cGZCII-VKK viruses (10^5^ TCID_50_ units, *n* = 5 or 6). PBS was used as a control. Mouse survival and disease development of each group were observed and scored until 15 dpi. The following scoring was used: healthy, 0 points; lethargy, inactivity, and limb weakness, 1 point; less exercise and limb paralysis, 2 points; quadriplegic and moribund, 3 points; and death, 4 points. (**E**) Pathological changes in different tissues from mice infected with cGZCII-98K, cGZCII-98K, and cGZCII-149V98K viruses (2 × 10^5^ TCID_50_) or PBS (as control) using the i.p. route. Mouse brains, lungs, livers, and hearts were harvested and subjected to histopathological examination at 3 dpi. Scale bar: 200 μm.

We further tested whether the enhanced cytotoxicity resulted in elevated virulence in mice. Two-week-old BALB/c mice were intraperitoneally inoculated with the EV71-GZCII or EV71 GZCII-P30 virus at a dosage of 10^4^ or 10^5^ TCID_50_ units. All mice infected with 10^5^ TCID_50_ units of the GZCII virus showed symptoms including lethargy, inactivity, limb weakness, limb paralysis, and ultimately death, within 5–9 days post-infection (dpi) (Figure 7A). In the 10^4^ TCID_50_ GZCII group, disease progression showed a 1-day delay, and the overall mortality decreased slightly (66.7%). However, GZCII-P30-infected mice survived the challenge experiment and showed no sign of disease even in the high-dose group, suggesting that GZCII-P30 was highly attenuated (Figure 7B).

We next infected mice with EV71-GZCII mutants with different residues at VP2-149, VP1-92, and VP1-98 to identify the determinant of the attenuation. Compared to the cGZCII virus, which rapidly (in five days) caused severe pathogenesis and death in all mice (6/6), the cGZCII-98K virus did not kill mice or cause any symptoms, and these effects were similar to those of the cGZCII-VKK virus (Figure 7C). cGZCII-149V92K, which contains two other mutations, killed 66.7% of the mice (four of six) and induced obvious disease (Figure 7D). These findings indicated that E98K alone attenuates the GZCII virus to a similar level as cGZCII-VKK and that the VP1-E98K mutation is responsible for virus attenuation in vivo.

Pathological analysis of GZCII mutant-infected mice (3 dpi) showed similar results. The cGZCII virus caused obvious lesions in mouse livers. Enlargement and severe disorganization of alveoli as well as pulmonary alveolar wall collapse were observed in cGZCII-infected mice (Figure 7E). The cGZCII-149V92K virus, which contains VP2-149V and VP1-92K mutations, caused similar pathogenicity in mouse livers and lungs. In contrast, the livers and lungs of GZCII-98K-infected mice showed no visible damages and were not different from those of the control mice (PBS group). Other tissues such as brainstems and hearts from all groups showed no obvious histopathological changes, indicating that the viruses did not cause damage in these areas at that timepoint.

### 3.8. HS and SCARB2 Are Distributed Differently in Mice

**Figure 8:**
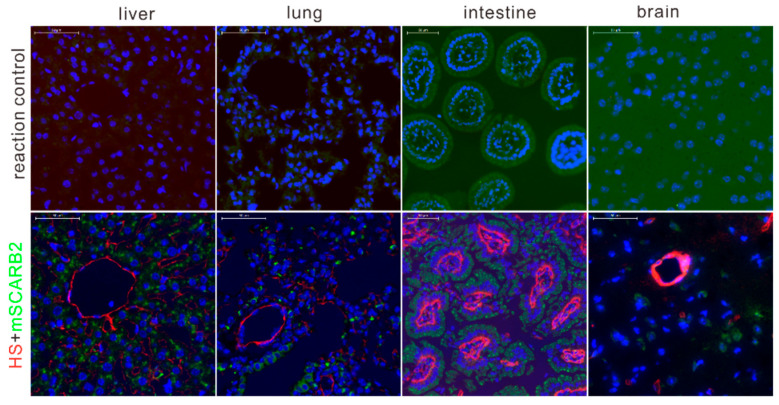
Expression of HS and SCARB2 proteins in different tissues of mouse. The liver, lung, intestine, and brain tissues were harvested from BALB/c mice. Sections incubated anti-HS and anti-SCARB2 antibody, or not incubated with primary antibodies (reaction control), were stained with fluorescent secondary antibodies to detect the expression of both receptors. Red: HS signal. Green: SCARB2 signal. Cell nuclei were stained with DAPI (blue). Scale bar: 50 μm.

HS and hSCARB2 have been reported to be differentially expressed in tissues of hSCARB2 transgenic mice [25], but their expression in wild-type mice remains unknown. We examined the expression and distribution of HS and mSCARB2 in the kidney, liver, intestine, and brain tissues of BALB/c mice through immunostaining (Figure 8).

In all examined tissue sections, HS was located mainly in blood vessels, whereas mSCARB2 was poorly expressed. In the liver, a strong HS signal was observed in vascular endothelial or sinusoidal endothelial cells, but not in other areas. However, SCARB2 was expressed in hepatocytes. In lung tissue, HS was distributed in the tracheal epithelium and vascular endothelial cells. In contrast, mSCARB2 was observed in other areas only. In the colon, SCARB2 was highly expressed near the serosa area, and HS was highly expressed in the lamina propria, which contained many capillaries. We also examined the expression patterns of HS and mSCARB2 in the brainstem, which is the major target area during EV71 infection in the CNS (Figure 8). SCARB2 expression was ubiquitously present in brain sections, but the cell types were not determined. In contrast, HS was poorly expressed in the brainstem, and HS-positive signals only appeared in blood vessel-like structures. SCARB2 was expressed in some HS-positive areas and showed some colocalization, but mostly in different areas (Figure 8). In summary, HS and SCARB2 expression in the mouse was different. The low level of colocalization of HS with SCARB2 indicated that the two receptors may not work cooperatively during EV71 infection in vivo.

### 3.9. E98K Mutation Changes the Tissue-Binding Activity of EV71 in Mice but Reduces Viral Replication In Vivo

**Figure 9:**
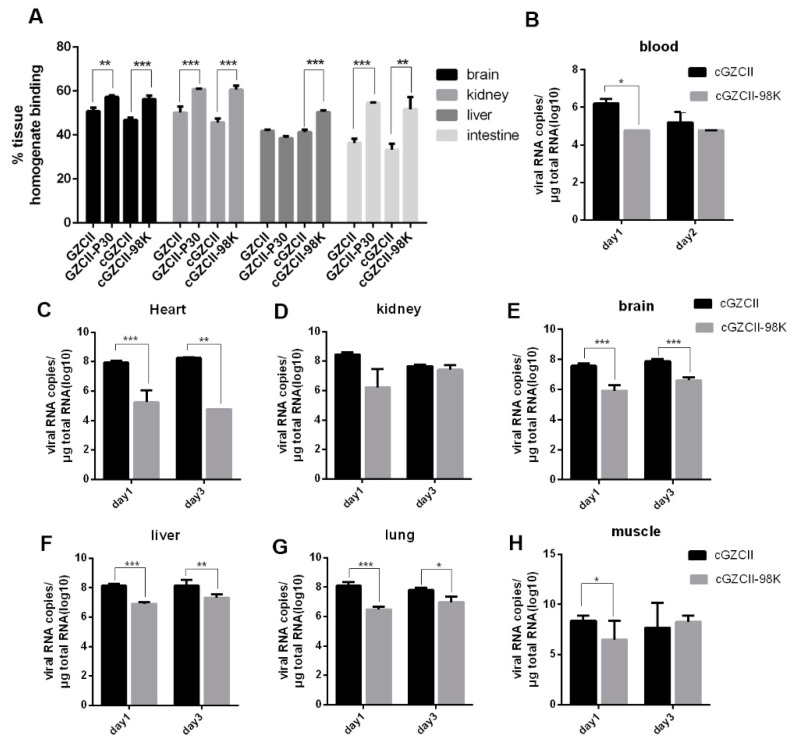
Binding efficiency and replication of GZCII variants in vivo. (**A**) Homogenates of mouse brain, kidneys, liver, or intestines (*n* = 3) were incubated with ~106 copies of GZCII variants for attachment, and the binding efficiency was determined by qPCR. (**B**) To monitor the viral load in mice, blood was collected at 1 dpi or 2 dpi, and mouse heart (**C**), kidney (**D**), brain (**E**), liver (**F**), lung (**G**), and muscle (**H**) tissues were harvested at 1 dpi or 3 dpi (*n* = 3). Viral RNA copy number in 1 μg of total RNA was determined by qPCR (*: *p* < 0.05; **: *p* < 0.01; ***: *p* < 0.001).

We proposed that the adsorption of cGZCII-98K by HS may be the reason for virus attenuation, as previously reported [25]. To confirm this hypothesis, we determined the binding efficiency of the GZCII virus with different mouse tissue homogenates. Four GZCII variants were mixed with tissue homogenates of mouse kidneys, liver, brain and intestines for attachment, and qPCR was performed to quantify the binding efficiency. All four EV71-GZCII variants showed greater than 36.4% binding to homogenates of mouse kidneys, liver, brain, and intestines. However, the binding ratios of GZCII-P30 and cGZCII-98K viruses were higher than those of wild-type GZCII and cGZCII viruses, and the differences (by Student’s t-test) were significant in most groups (Figure 9A). These results were similar with those of the cell-binding and HS-binding experiments.

We also determined the distribution of cGZCII and cGZCII-98K viruses in different tissues or blood from infected mice. In mouse heart, kidney, liver, lung, brain, and muscle tissues (Figure 9B–H), viral RNA copies of cGZCII were much higher than those of cGZCII-98K at 1 dpi and 3 dpi. Significant differences (>1 log higher, *p* < 0.05) were observed in mouse brain, heart, liver, and muscle tissues at both 1 dpi and 3 dpi. In blood collected from cGZCII- or cGZCII-98K-infected mice at 1 dpi or 2 dpi, cGZCII viral RNA copies were also higher than those of cGZCII-98K. For the cGZCII-98K virus, less than 10^5^ copies of the cGZCII-98K virus were detected in the blood, although >10^6^ copies of viral RNA/μg total RNA could be detected in most mouse tissues at 1 dpi. These results suggested that the strong binding activity in mouse tissues resulted in impaired replication of GZCII-98K virus in vivo, which may account for the attenuated virulence.

## 4. Discussion

The L929-adapted strain, EV71 GZCII-P30, showed a significantly enhanced replication ability in two mouse-derived cell lines, namely, L929 and Neuro2a (Figure 1). The distinct phenotype represented the fitness of the resulting GZCII-P30 virus in murine cells. The EV71 virus cannot bind to mouse SCARB2, and L929 is poorly permissive to EV71 [26]. The success in developing the L929-adapted EV71 strain may help to understand the molecular mechanism underlying virus adaptation in mice.

NGS analysis revealed three mutations on the GZCII-P30 viral capsid compared to the GZCII parental strain capsid (Figure 2). VP2-149 variations have been identified in multiple mouse-adapted [27,28] or murine cell line-adapted EV71 strains [13,29], implying its importance for binding to PSGL-1 or mSCARB2 [30]. However, whether mSCARB2 can mediate EV71 infection has not been determined. Our study using mSCARB2-KO cells revealed the dependency of EV71-GZCII on mSCARB2 for virus infection (Figure 4). Considering the high enrichment, the roles of VP2-I149V warrant further study.

VP1-E98K increased the virus titer to a level comparable to that of the GZCII-P30 virus, while the other two mutations only slightly enhanced viral replication (Figure 3). Victorio et al. reported that a murine cell-adapted strain of EV71:BS, which carries K98E, E145A, and L169F, shows improved replication in murine cell lines [13], and these results contradict the present findings. However, viral antigens were detected in only NIH3T3 cells inoculated with clone-derived BS-VP1^L169F^ and BS-VP1^E145A^ viruses, but not BS-VP1^K98E^ [13], indicating that the K98E mutation may not contribute to the adaptation of the BS variant to murine cells [13], and the compensation effect of VP1-E145A on the VP1-K98E mutation should also be considered. Our results suggested that VP1-E98K is the key factor for GZCII adaptation.

More GZCII-P30 and cGZCII-98K viruses attached to L929 cells and HS than GZCII or cGZCII viruses, and the VP1-E98K mutation was found to be responsible for the enhanced attachment (Figure 5). GZCII-P30 and cGZCII-98K viruses were also more sensitive to HS-depletion by EXT1-knockout or heprarinase treatment (Figure 6). The GZCII-P30 virus consisted of diverse variants (see NGS data), this may explain why it was more affected by HS-depletion than the cloned cGZCII-98K virus in some cases. Together, these results indicate that VP1-E98K may promote viral replication in cultured cells by strengthening virus binding to cell surface HS. Another research, which was published during the preparation of this manuscript, also found that the cell culture adaptation of EV-A71 is due to HS binding [31]. The low titer of cGZCII-98K and GZCII-P30 detected in mSCARB2-KO cells (Figure 4C) may represent viruses that had bound via HS from the beginning, Interestingly, three other lysine residues, which have been identified as critical for HS binding, namely, K162, K242 and K244 [14], all existed in the GZCII strain. These residues may explain the innate HS-binding ability and the sensitivity of the EV71-GZCII and cGZCII viruses to soluble HS or HS depletion (Figure 5 and Figure 6).

Contrary to cell infection experiments, the HS-binding property was negatively associated with viral virulence and pathogenicity (Figure 7) in mice. The improved HS-binding activity of the GZCII-E98K virus resulted in decreased, rather than enhanced, replication in mouse tissues and blood (Figure 9). Detection of the expression and distribution of HS and SCARB2 in mice showed that HS was highly expressed in blood vessel-like structures, where SCARB2 was expressed at a low level (Figure 8). These results suggest that HS and SCARB2 may not work cooperatively during EV71 infection and that the VP1-98 residue may regulate virulence and pathogenicity through HS binding.

Rapid trapping of the EV71-145G virus by HS-rich tissue and the consequent prevention of viremia are considered factors of viral attenuation in vivo [25,32,33]. A recent study also reported that VP1-98E and other poor HS-binding viruses are highly lethal in mice, resulting from better in vivo dissemination [17]. Similar mechanisms have been revealed for a wide range of viruses including flaviviruses [34,35,36,37], togaviruses [38,39,40,41,42], picornaviruses [43,44], and some DNA viruses [45]. In our study, the strong ability to bind to mouse tissue homogenates and the relatively low viral load in multiple tissues of E98K virus-infected mice also suggest HS-dependent attenuation. However, a lower level viremia of the 98K mutant was only observed on day 1 (Figure 9B). A thorough viral kinetics in the blood would help to reveal the role of HS-binding activity on EV71 dissemination. Another important limitation of our study is that the HS-binding activity and replication kinetics of the EV71 variants were only analyzed in L929 cells. Using clinic relevant cell lines such as microvascular endothelial cells or brain-derived cells from human or mouse will help to explain the relationship between HS-binding activity and virulence of the EV71 VP1-98K mutant.

Analysis of the EV71 sequence in clinical samples has indicated that E98K and E145G, which represent the HS-binding phenotype, are frequently associated with severe neurological disease in humans [46,47,48]. The HS-binding ability resulting from VP1-L97R has been attributed to the improved dissemination of an EV71 strain in an immunosuppressed patient [15,49]. These studies contradicted our conclusions. As EV71 viruses are generally injected directly into animals intraperitoneally or intravenously, the lack of selection by the respiratory or intestinal barrier during natural infection in humans may explain the difference, as pointed out in [15,17].

## 5. Conclusions

We demonstrated that the E98K mutation is responsible for the adaptation of a virulent mouse EV71-GZCII virus to murine cells in vitro as well as its attenuation in mice. It has been proposed that HS-independent EV71 viruses are highly resistant to neutralizing antibodies, which may affect EV71 virulence [32]. In this study, we only focused on the difference in GZCII variants in their cellular HS-binding ability. Further study of their resistance to antibody neutralization may help to fully elucidate the reason for their distinct pathogenicity. The HS-binding ability associated with the E98K mutation may accelerate viral replication in the presence of the SCARB2 receptor in vitro, but trapping viruses with low SCARB2 expression in mice may lead to impaired pathogenesis. Our study may help to unravel the association of EV71 virulence with its receptor usage, providing insights into viral attenuation.

## Supplementary Materials

The following are available online at https://www.mdpi.com/1999-4915/12/8/883/s1, Figure S1, Sequence analysis of the constructed GZCII variants; Table S1, DNA oligos used in this study.
