# Supplementary material for: A Single Mutation in the VP1 Gene of Enterovirus 71 Enhances Viral Binding to Heparan Sulfate and Impairs Viral Pathogenicity in Mice
Source: Viruses. 2020 Aug 13;12(8):883. doi: 10.3390/v12080883 (PMC7472116; doi:10.3390/v12080883)
Supplement: Supplementary file 1 [file viruses-12-00883-s001.pdf]

Supplementary Information

Figure S1

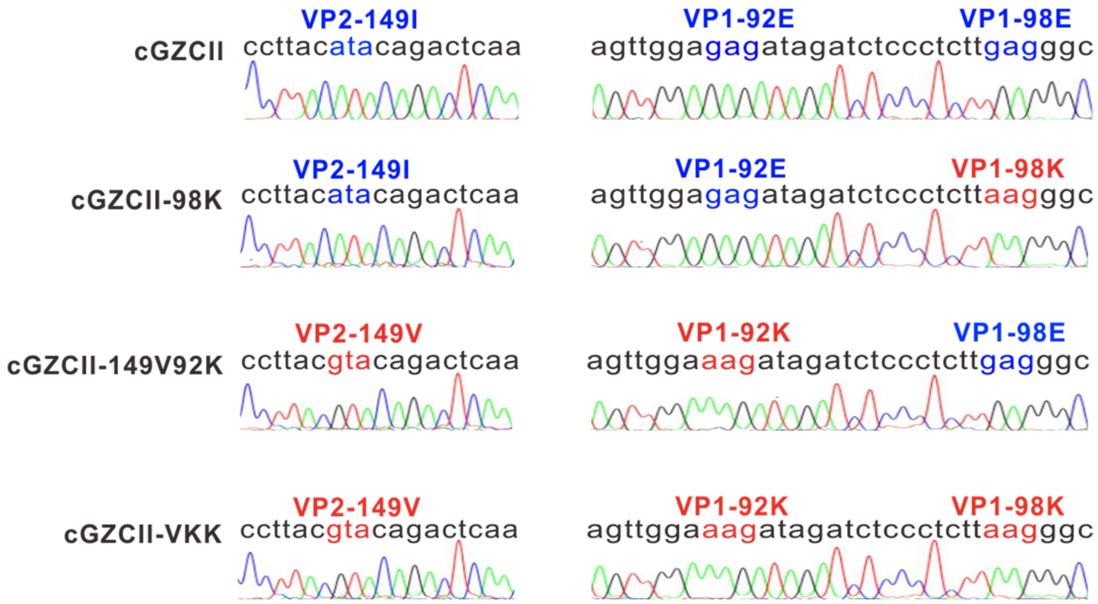

Figure S1. Sequence analysis of the constructed GZCII variants with different mutations at the VP2-149, VP1-92 and VP1-98 residues. The VP1 and VP2 regions were amplified from viral cDNA and subjected to Sanger sequencing to confirm mutagenesis.

Table S1. DNA oligos used in this study

Oligos	Sequence	Application
**Vec-F**	gggggtaaatttgttataaccagaatagca	Construction of pEV71-GZCII
**Vec-R**	ccacaggctttttaacctatagtgagtcgtattaatacgtaatttcg	
**GZCII-1F**	ttaaaacagcctgtgggttgcacccactc	
**GZCII-3470R**	acaaccttgggcggtggtggat	
**GZCII-3470F**	accgcccaaggttgtgatactattgcc	
**GZCII-7404R**	aacaaatttacccccaccagtcacattcacg	
**149V-F**	ccccccttacgtacagactcaacccggtgccgatg	Site-directed mutagenesis to construct recombinant EV71-GZCII viruses
**149V-R**	ctgtacgtaaggggggtgggtgtcttccg	
**92K-F**	ttagttggaaagatagatctccctcttgagggcacaact	
**92K-R**	tatctttccaactaatccggccctgctgaaga	
**98K-F**	cctcttaagggcacaactaacccgaatggt	
**98K-R**	tgtgcccttaagagggagatctatctctccaactaatccggc	
**92K98K-F**	ttagttggaaagatagatctccctcttaagggcacaact	
**92K98K-R**	tatctttccaactaatccggccctgctgaaga	
**GZCII-Seq-1F**	ttaaaacagcctgtgggttgcacc	Preparation of 2 DNA fragments covering the entire genome for NGS
**GZCII-Seq-1R**	cttgtgaggcagcagattgttcc	
**GZCII-Seq-2F**	cgacatggttaccctcactgca	
**GZCII-Seq-2R**	gctattctggttataacaaatttacccccacc	
**sgEXT1-S**	caccggacccaggcaggacacatgc	sgRNA expression plasmid construction
**sgEXT1-NS**	aaacgcatgtgtcctgcctgggtcc	
**sgSRB2-S**	caccgagatgctgcttctacacggc	
**sgSRB2-NS**	aaacgccgtgtagaagcagcatctc	
**SRB2KOid-F**	ctctgctgctcattccggtatct	Amplification of the sgRNA target region for genotyping
**SRB2KOid-R**	gcaaacaagagctgtgttctgca	
**EXT1KOid-F**	cccacatgcgctggatcagt	
**EXT1KOid-R**	gtgcacgctggaatcctcgt	
**qEV-F**	aaatggcagcgccactgaag	qRT-PCR detection of EV71 RNA copies
**qEV-R**	gggcgcgttggtttatccac	
**qEV probe**	FAM-tccaccatcaccacgcaagaagcggct-TAMRA	
**mGAPDH-F**	aggtcggtgtgaacggatttg	qPCR detection of the mouse GAPDH gene
**mGAPDH-R**	tgtagaccatgtagttgaggtca	
**Vec-F**	gcagctgtggaatgtgtgtcagt	Sequencing of pEV71-GZCII
**630-R**	caccggatggccaatcca	
**1310-R**	aggagtgctccttggtgg	
**2030-R**	gggtgtagtatccgcacaac	
**2750-R**	gttggcataaccattcgggt	

**3470-R**	acaaccttgggcggtggtggat	
**4110-R**	tagcggcgttggccatatc	
**4820-R**	agtctgtcacttcaatgtcac	
**5530-R**	tcacgagtttgtgctcaatcc	
**6230-R**	caggcctcctccatgctc	
**6930-R**	gggatagctagcgagcacatc	
**7570-R**	ggttattgtctcatgagcgga	
